# Severe complications at the interface of psychiatry and intensive care: Clinical profile of transferred patients

**DOI:** 10.1192/j.eurpsy.2026.10761

**Published:** 2026-07-31

**Authors:** V. Tapes, I. Cosciug, I. Deliv

**Affiliations:** Mental Health, Clinical Psychology and Psychotherapy Department, University of Medicine and Pharmacy “Nicolae Testemitanu”, Chisinau, Moldova, Republic of

## Abstract

**Introduction:**

Mental disorders can evolve unpredictably, sometimes progressing to acute states that pose significant risks to the patient. Such acute episodes due to a somato-psychic neurovegetative process require urgent intervention, intensive monitoring, and multidisciplinary therapeutic approaches, given their potential for rapid deterioration of the patient’s general condition. Admission to intensive care units (ICUs), although relatively rare, signals major decompensation, reflecting the complexity and severity of the pathological process.

**Objectives:**

This study will investigate the acute somato-psychiatric profile requiring transfer to the ICU from psychiatric wards, with the aim of identifying factors of severe decompensation, underlining the complexity of the clinical evolution and highlighting the challenges of multidisciplinary interventions in psychiatric emergencies.

**Methods:**

The study was conducted retrospectively based on clinical observation records of patients admitted to psychiatric wards and subsequently transferred to the ICU between 2022 and 2025. Demographic data (age, gender), main psychiatric diagnoses, somatic comorbidities, and discharge status were collected and analyzed. Diagnoses were classified according to ICD-10 criteria, and data were stratified by age group (<18 years, 19–44 years, 45–64 years, and ≥65 years) to highlight distribution and clinical characteristics.

**Results:**

The patient cohort included individuals aged between 14 and 79 years, of whom 4.0% were <18 years,19-44 years (26.0%), 45-64 years (45.0%), and 25.0% ≥65 years. The gender distribution showed a male predominance (60.0%) compared to females (40.0%). In patients <18 years of age, autism spectrum disorders, acute psychotic episodes, and severe depressive disorders with psychotic elements predominated, frequently associated with malnutrition, gastroesophageal reflux with esophagitis, and iron deficiency anemia, with no cases of death. Whereas in those aged ≥18 years, the dominant psychiatric diagnosis was paranoid schizophrenia (≈48%), followed by catatonic schizophrenia (≈11%), organic and behavioral disorders (≈10%), dementias (≈9%) and acute psychotic disorders with symptoms of schizophrenia (≈7%), associated with ischemic cardiomyopathy, congestive heart failure, multiple organ failure, pulmonary tuberculosis, chronic hepatitis, respiratory infections (including SARS-CoV-2), diabetes mellitus, and nutritional disorders. Mortality in the ICU was ≈10%.

**Conclusions:**

The admission of patients with psychiatric disorders to ICU wards is due to both severe mental decompensation and the complexity of associated somatic comorbidities. Early identification of risk factors, continuous monitoring, and interdisciplinary collaboration are essential elements for optimizing outcomes and implementing individualized management strategies for this category of patients.

**Disclosure of Interest:**

None Declared

